# Social health in young women with chronic pain

**DOI:** 10.1097/PR9.0000000000001146

**Published:** 2024-03-18

**Authors:** Ian A. Boggero, Linda Sangalli, Lauryn Brasch, Christopher D. King

**Affiliations:** a Division of Orofacial Pain, Department of Oral Health Science, College of Dentistry, University of Kentucky, Lexington, KY, USA; b Department of Psychology, College of Arts and Science, University of Kentucky, Lexington, KY, USA; c Department of Anesthesiology, College of Medicine, University of Kentucky, Lexington, KY, USA; d College of Dental Medicine—Illinois, Midwestern University, Downers Grove, IL, USA; e Division of Behavioral Medicine and Clinical Psychology, Department of Pediatrics, Cincinnati Children's Hospital Medical Center, Cincinnati, OH, USA; f Department of Pediatrics, University of Cincinnati College of Medicine, Cincinnati, OH, USA; g Pediatric Pain Research Center (PPRC), Cincinnati Children's Hospital Medical Center, Cincinnati, OH, USA

**Keywords:** Social functioning, Overlapping chronic pain conditions, Young adults, Social isolation, Social roles and activities

## Abstract

**Introduction:**

Chronic pain may negatively affect social functioning, but no study to date has examined the specific social impact of different chronic pain conditions in young women, and whether living with multiple chronic overlapping pain conditions (COPCs) differently influences social domains.

**Objectives:**

This study aimed to assess social functioning (social isolation, hostility, informational support satisfaction, social roles, emotional support, friendships, and family relationships) among young women with chronic pain compared with pain-free controls and to test whether the number of COPCs influenced the extent of social burden.

**Methods:**

Participants aged 18 to 30 years with a physician-confirmed diagnoses of migraine, fibromyalgia, or temporomandibular disorder (TMD) and pain-free controls were invited to participate from across the United States. After confirming eligibility, participants completed a 1-hour REDCap online questionnaire assessing social functioning.

**Results:**

One hundred four participants (mean age 24.54 ± 3.35 years) were included (n = 26 with TMD, n = 25 with fibromyalgia, n = 25 with migraine, and n = 28 controls). All 3 chronic pain groups combined reported worse functioning than controls on friendship (*P* = 0.038), social isolation (*P* = 0.002), and social roles (*P* < 0.001). There were no differences on social variables between the 3 chronic pain groups (all *P'*s > 0.05). Compared with those with 3 COPCs, participants with 1 condition reported better family relationships (*P* = 0.024).

**Conclusions:**

Experience of chronic pain—regardless of the specific pain condition—may negatively affect some areas of social functioning in young women.

## Abstract 2

The experience of chronic pain—regardless of the specific condition—may negatively affect friendships, social isolation, and satisfactions with social roles/activities in young women.

## 1. Introduction

The biopsychosocial model of chronic pain posits that pain is affected by interactions between biological, psychological, and social factors. Yet, chronic pain research has disproportionately focused on biological/psychological factors, with fewer studies examining social correlates of pain.^1,3,11,17,20,21,26,27,32,37,39,43,50–52,69^ Extant studies show that relative to pain-free populations, individuals with chronic pain report lack of friendships and feelings of social isolation,^26,43,55,67,68,75^ more victimization/isolation,^31,32^ greater dissatisfaction with social roles/activities,^32,73^ and poor emotional support,^32,72^ among other negative social outcomes.^28,32,46,68^ In addition, youth with chronic pain experience developmental delay in education,^51,52^ relationships,^31^ and autonomy^21^ and are less likely to attend social/family events,^13,20,57,70^ suggesting that chronic pain may negatively affect multiple social domains.

The impact of pain on social functions may be exacerbated in those with multiple pain conditions. Chronic overlapping pain conditions (COPCs) are a set of commonly comorbid disorders, including temporomandibular disorders (TMD), fibromyalgia, and migraine, among others that share overlapping physiological, genetic, and pain mechanisms.^4,22,30,53,56,59,62^ COPCs are associated with worse psychological function in a gradient-specific manner, based on the number of overlapping conditions.^59^ Although research has examined sociopsychological and biological variables of these chronic pain conditions alone,^30^ no work, to our knowledge, has examined the broad social impact of COPCs.

Moreover, social impact of pain may not be similar across the life span. One in 9 young adults experience chronic pain worldwide,^58^ and young adulthood (ages 18–30 years) is a crucial period of life for development of personal identity and lifelong social bonds.^2,6,39,77^ Previous research has found that female individuals are more likely than male individuals to seek treatment for several chronic pain conditions,^8,36^ suggesting that they may be the most socially affected by pain. Although others have called for explicitly examining the needs of young adults with chronic pain specifically, relatively few studies have specifically examined social parameters in this population.^14,74^ Thus, investigating the impact of pain on social functioning in this developmental period is imperative.

The aim of this study was to compare social functioning among young women (ages 18–30 years) with TMD, fibromyalgia, migraine, and a combination thereof (ie, COPCs), and healthy controls. First, we will test which specific social domains (Table 1) are different between young women with chronic pain and controls. Second, we will test whether social impact differs among those with 3 different pain conditions. Temporomandibular disorders consist of pain in the head/face and may have a substantial impact on social functioning and activities because pain is aggravated by jaw function (chewing, speaking, and talking) and expressing emotions (smiling and laughing^40^). Migraine consists of pain in the head, which is *not* triggered by social activities, providing a good comparison group. Fibromyalgia is characterized by generalized bodily pain, allowing us to test whether pain in the head/face is particularly disruptive. Finally, we will test the impact of COPCs on social functioning among young women. We hypothesized that young women in all chronic pain groups would report worse social functioning than controls (Aim 1). Because social activities would likely aggravate TMD pain, we hypothesized that social outcomes would be more impaired in those with TMD (Aim 2). Finally, we hypothesized that young women with COPCs would report worse social functioning than those with only 1 pain condition (Aim 3). To the best of our knowledge, this is the first study to comprehensively characterize social health parameters in young women with COPCs.

**Table 1:** Definition and sample items for social functioning battery.

Social construct	Definition	Sample items
Emotional support	The perception that people in one's social network are available to listen to one's problems with empathy, caring, and understanding	“*In the past 4 weeks, describe how often I have someone who will listen to me when I need to talk*” “*In the past 4 weeks, describe how often I have someone to confide in or talk to about myself or my problems*”
Family relationships	Frequency of normal family routines, effectiveness of family communication and problem solving, family cohesiveness, and how well family members get along	“*In the past 4 weeks, I felt I had a strong relationship with my family*” “*In the past 4 weeks, I felt really important to my family*”
Friendship	Perceptions of the availability of friends or companions with whom to interact or affiliate	“*In the past 4 weeks, I got invited to go out and do things with other people*” “*In the past 4 weeks, I have friends I get together with to relax*”
Informational support	The perception that people in one's social network are available to provide material or functional aid in completing daily tasks, if needed	“*In the past 4 weeks, described how often I have someone to give me good advice about a crisis if I need it*” “*In the past 4 weeks, described how often I have someone to turn to for suggestions about how to deal with a problem*”
Social isolation	Perceptions that one is alone, lonely, or socially isolated from others	“*I feel left out*” “*I feel that people barely know me*”
Social roles and activities	Satisfaction with performing one's usual social roles and activities	“*I am satisfied with my ability to do things for my family*” “*I am satisfied with my ability to do things for fun with others*”
Hostility	The extent to which an individual perceives his/her daily social interactions as negative or distressing. This can include aspects of perceived hostility	“*In the past month, described how often people in your life argue with me*” “*In the past month, described how often people in your life act in an angry way toward me*”

## 2. Methods

### 2.1. Recruitment and eligibility

This study was conducted entirely online and by phone. Participants were recruited nationwide through ResearchMatch and through online advertisements/emails sent to chronic pain listservs. The recruitment process lasted 24 months (October 2019–February 2021). To be eligible, participants needed to be female, be between 18 and 30 year of age, be on a stable medication regimen during the previous 4 weeks, understand English, and have internet access. Participants were excluded if they self-reported flu-like symptoms within the previous 2 weeks, current use of opioids, being pregnant, or being diagnosed with cancer. To qualify for the chronic pain group, patients also needed a positive chronic pain screen (see Materials section below) and a physician-confirmed diagnosis of TMD, migraine, or fibromyalgia. Healthy controls were included if they had a negative pain screen and no lifetime history of chronic pain. We first recruited the 3 chronic pain groups (simultaneously) and then recruited the healthy women after sufficient participants were obtained for the pain groups.

### 2.2. Study procedures

Interested participants were instructed to call the study coordinator at Cincinnati Children's Hospital Medical Center. A research coordinator confirmed eligibility criteria over the phone, asked potential participants about their previous physician-confirmed chronic pain diagnosis, and conducted a brief screener of TMD, fibromyalgia, and chronic migraine. To be eligible, participants in any of the 3 chronic pain groups needed to screen positive on 1 or more of these screeners and report having a physician-confirmed diagnosis for that same condition, by answering yes to the question “Have you ever been diagnosed by a medical professional with fibromyalgia, migraine, or temporomandibular joint disorder or other chronic pain condition in the face?” If participants scored positive and had a physician-confirmed diagnosis for 2 or more conditions, they were asked which of the conditions was most disruptive to their day-to-day functioning and were placed in that group. Participants in the healthy control group needed to screen negative on all 3 screeners and report a lifetime absence of any chronic pain condition.

After completing the 15-minute phone call with the study members, eligible participants were e-consented. Participants completed all aspects of the study from their home (or anywhere else they had internet access). They were then emailed a link to a REDCap questionnaire containing the pain and social measures described further. They were told that the questionnaire would take approximately 1 hour to complete and that they were given 7 days to complete it before the survey link expired to prevent completion outside of the 7-day window. The participants who completed the questionnaire were mailed a $15 Target gift card. The study was approved by the IRB at Cincinnati Children's Hospital (IRB#2019-0855).

### 2.3. Materials

#### 2.3.1. Telephone screening instruments

##### 2.3.1.1. Temporomandibular disorder screener

Participants were assessed for the presence of TMD using a 3-item questionnaire.^33^ They were asked about the duration of pain in the jaw or temple in the past 30 days (0 = “no pain,” 1 = “from very brief to more than a week, but it does stop,” and 2 = “continuous”), the presence of jaw stiffness upon awakening (0 = “no” and 1 = “yes”), and whether they experienced changes in pain with jaw function (0 = “no” and 1 = “yes”). A total score was calculated by summing all items, and a score ≥3 was considered a positive screen for TMD.

##### 2.3.1.2. Chronic migraine screener

Participants were assessed for the presence of migraine with a 12-item questionnaire.^48^ Questions 1 to 2 assessed the frequency of headache in the past 90 and 30 days, respectively. Questions 3 to 6 assessed the presence of associated symptoms (ie, nausea, photophobia, and phonophobia) and headache severity in the past 30 days (0 = “never,” 1 = “rarely,” 2 = “less than half the time,” and 3 = “more than half the time”). Questions 7 to 12 assessed the use of medications, work/school or social activity absenteeism, and headache interference in the past 30 days. A positive screen for migraine was defined by the presence of associated symptoms and high headache frequency or by high medication intake and absenteeism and interference.^48^

##### 2.3.1.3. Fibromyalgia screener

Participants were assessed for fibromyalgia using 6 items.^54^ The first question measured the Widespread Pain Index (WPI) by assessing the presence of pain in 19 body sites in the previous week (0 = “no” and 1 = “yes”). Questions 2 to 5 measured the Symptom Severity Index (SSI), which assessed fatigue severity, feeling unrefreshed on awakening, lack of concentration over the past week, and presence of headache, abdominal pain, and depression. The SSI yielded a score between 0 and 12. Question 6 confirmed a symptom duration of more than 3 months. A total score of 13 or above on the summed WPI and SSI was considered a positive screen for fibromyalgia.^76^

#### 2.3.2. REDCap assessment

Participants completed the following social outcome battery through REDCap. Although this was the parent study, we also collected additional measures assessing psychological functioning, fatigue, and sleep for exploratory analyses that are outside the scope of this study.

##### 2.3.2.1. Demographics

Participants self-reported their age, marital status, race, annual household income, and current use of medication (yes/no).

##### 2.3.2.2. Medication

For descriptive purposes, participants were asked to report their current prescription medications, along with the reason for which they were prescribed, and the doses for each medication.

##### 2.3.2.3. Pain intensity

The average pain intensity was assessed by a single item asking participants as follows: “please rate your pain by marking the box with the number that best describes your pain on the AVERAGE” using a scale of 0 = “no pain” to 10 = “worst pain imaginable.” This single item was drawn from the Brief Pain Inventory, which is widely used and well-validated for assessing pain intensity in populations with chronic pain.^16^

##### 2.3.2.4. Social outcomes

All social outcomes assessed for this study were from the PROMIS/NIH Toolbox measures, which are validated in individuals with chronic conditions.^17,18,35^ Sample items of each measure are summarized in Table 1. When different versions of the same scale were available, we used the longer version to maximize reliability. We chose one of each of the short-form measures available except for companionship because it seemed to closely overlap with social isolation/loneliness and instrumental support because several of the items did not seem appropriate for a young adult population.

###### 2.3.2.4.1. Social isolation

The social isolation measure consisted of 8 items rated on a scale of 1 = “never” to 5 = “always,” with higher scores indicating greater perceived social isolation. A total score was calculated by summing all items. The value of Cronbach alpha (α) is 0.93.^6^ This measure has been used to assess social isolation in young adults in the United States.^60^

###### 2.3.2.4.2. Family relationship

The pediatric family relationship measure consisted of 8 items rated on a scale of 1 = “never” to 5 = “always,” with higher scores indicating greater support from family relationships. A total score was calculated by summing all items (*α* = 0.94). An adult version of this instrument was unavailable. However, previous work has shown that family relationships are a critical component of quality of life for young adults with chronic health conditions,^61^ and as such, we wanted to assess whether family relationships differed between the chronic pain categories assessed in this study.

###### 2.3.2.4.3. Informational support

The informational support measure consisted of 8 items rated on a scale of 1 = “never” to 5 = “always,” with higher scores indicating greater perceived informational support. A total score was calculated by summing all items (*α* = 0.91).

###### 2.3.2.4.4. Friendship

The friendship measure consisted of 8 items rated on a scale of 1 = “never” to 5 = “always,” with higher scores indicating greater friendship support. A total score was calculated by summing all items (*α* = 0.90). This measure has been used to assess perceptions of friendship in young adults.^47^

###### 2.3.2.4.5. Satisfaction with social roles and activities

The satisfaction with social role measure consisted of 8 items rated on a scale of 1 = “not at all” to 5 = “very much,” with higher scores indicating greater satisfaction with social roles. A total score was calculated by summing all items (*α* = 0.92). This scale has been used to assess satisfaction with social roles in young adults with chronic health conditions.^45^

###### 2.3.2.4.6. Emotional support

The emotional support measure consisted of 8 items rated on a scale of 1 = “never” to 5 = “always,” with higher scores indicating greater perceived emotional support. A total score was calculated by summing all items (*α* = 0.96). This measure has been used to assess emotional support in young adults in the United States.^66^

###### 2.3.2.4.7. Hostility

The hostility measure consists of 8 items rated on a scale of 1 = “never” to 5 = “always,” with higher scores indicating greater hostility. A total score was calculated by summing all items (*α* = 0.92). This measure has previously been used to assess perceived hostility in young adults with and without chronic health conditions.^49^

### 2.4. Statistical analysis

Before analyses, variables were checked for missing information and outliers (±3 SD, from the mean). Missing data were left missing and removed from analyses on a case-wise basis. When outliers were identified, analyses were run with and without the outlier. If removing the outlier changed the results substantively, models with and without the outlier are reported further. If removing the outlier did not change the results, then outliers were retained to maximize power.

Aim 1 was to test whether young women with chronic pain differed from healthy controls regarding social outcomes. The 3 chronic pain groups (TMD, fibromyalgia, and migraine) were combined and coded 1 for the chronic pain group and 0 for the pain-free controls. Comparisons between the chronic pain and pain-free groups in terms of social functioning were performed with independent *t* tests. Because the control group was smaller than the pain group, we ran Levene test to see whether variances were similar between groups. Results revealed equal variances on all variables except for satisfaction with social roles and activities. However, the interpretation did not change when adjusting for the unequal variances between groups for that variable, so results presented are for models assuming equal variances between groups.

Aim 2 was to compare social functioning between young women experiencing TMD, fibromyalgia, and chronic migraine. Analysis of variance was used to compare the 3 groups, with Tukey post hoc tests. Models were then rerun using ANCOVA to control for pain intensity as a covariate. Effect size comparisons between the groups were computed using Cohen D.

Aim 3 was to compare social functioning between those with none vs only 1 vs 2 vs all 3 chronic pain conditions. To do this, we created a new variable coded 0 for healthy controls, 1 for those with only 1 chronic pain condition as determined by a positive screener, 2 for those who screened positive for any 2 chronic pain conditions, and 3 for those who screened positive for all 3 conditions. ANCOVA was used to compare the 4 groups, and significant relationships were explored with Tukey post hoc tests. Models were run with and without controlling for pain intensity as a covariate. Analysis of variances were used to compare age and income between those with different number of pain conditions.

For all analyses, the *P* value was set at < 0.05. Data were analyzed with SPSS (IBM SPSS Statistics Macintosh, Version 27.000, IBM Corp, Armonk, NY).

## 3. Results

Several hundred people were notified of the study on ResearchMatch. Of them, 155 expressed expressing interest in participating in the study. One hundred ten underwent a phone screening and were deemed eligible. Six did not start or complete the online questionnaires and were thus excluded. Thus, the final sample consisted of 104 people for analysis. Twenty-six were in the TMD group, 25 in the fibromyalgia group, 25 in the migraine group, and 28 in the pain-free group. Demographic characteristics are summarized in Table 2.

**Table 2:** Demographic and social functioning differences between young women with temporomandibular disorder, fibromyalgia, and migraine and pain-free controls.

	Pain-free group (N = 28)	Chronic pain group				*P* *	*P* †	*P* ‡	Cohen *D* comparing pain-free group with chronic pain group (combined)
		Total (N = 76)	TMD (N = 26)	Fibromyalgia (N = 25)	Migraine (N = 25)				
Age (y), mean ± SD	24.00 ± 3.24	24.74 ± 3.38	23.73 ± 3.38	25.56 ± 3.78	24.96 ± 2.79				
Marital status (%)									
In a relationship, not married	5 (17.85%)	23 (30.27%)	11 (42.31%)	3 (12.00%)	9 (36.00%)				
Married	4 (14.29%)	11 (14.47%)	4 (15.38%)	3 (12.00%)	4 (16.00%)				
Divorced	0 (0.0%)	2 (2.63%)	0 (0.0%)	0 (0.0%)	2 (8.00%)				
Single	19 (67.86%)	40 (52.63%)	11 (42.31%)	19 (76.00%)	10 (40.00%)				
Race (%)									
African American	1 (3.57%)	0 (0.00%)	0 (0.00%)	0 (0.00%)	0 (0.00%)				
Caucasian	27 (96.43%)	76 (100%)	26 (100%)	25 (100%)	25 (100%)				
Income (%)									
Less than $19,999	1 (3.57%)	9 (11.84%)	5 (19.23%)	2 (8.00%)	2 (8.00%)				
$20,000–$39,999	5 (17.86%)	16 (21.05%)	6 (23.08%)	7 (28.00%)	3 (12.00%)				
$40,000–$59,999	8 (28.57%)	15 (19.74%)	1 (3.85%)	7 (28.00%)	7 (28.00%)				
$60,000–$79,999	4 (14.29%)	8 (10.52%)	2 (7.69%)	3 (12.00%)	3 (12.00%)				
$80,000–$99,999	1 (3.57%)	6 (7.89%)	1 (3.85%)	3 (12.00%)	2 (8.00%)				
$100,000–$149,999	3 (10.71%)	11 (14.49%)	4 (15.38%)	2 (8.00%)	5 (20.00%)				
Higher than $150,000	4 (14.29%)	3 (3.95%)	2 (7.69%)	0 (0.0%)	1 (4.00%)				
Do not know	2 (7.14%)	8 (10.52%)	5 (19.23%)	1 (4.00%)	2 (8.00%)				
Social functioning, mean (SD)									
Emotional support	34.64 (7.29)	34.01 (5.91)	33.77 (6.50)	33.96 (6.51)	34.32 (4.77)	0.65	0.95	0.72	
Family relationships	31.89 (7.48)	29.07 (7.23)	29.19 (7.23)	29.04 (7.97)	28.96 (6.75)	0.08	0.99	0.95	
Friendship	28.96 (7.29)	25.50 (7.53)	26.23 (8.52)	24.20 (6.64)	26.04 (7.40)	**0**.**038**	0.58	0.74	0.34
Informational support	33.82 (5.05)	32.38 (4.92)	32.73 (4.44)	32.16 (4.71)	32.24 (5.71)	0.19	0.91	0.76	
Social isolation	17.89 (4.01)	22.39 (6.81)	23.12 (8.17)	21.28 (5.18)	22.76 (6.83)	**0**.**002**	0.60	0.37	0.81
Social roles	32.04 (4.62)	25.47 (6.88)	26.92 (7.38)	24.56 (5.68)	22.88 (7.43)	**<0.001**	0.42	0.52	1.12
Hostility	14.04 (5.22)	15.80 (5.63)	16.77 (6.58)	16.16 (5.17)	14.44 (4.93)	0.16	0.32	0.38	
Pain intensity, mean ± SD	n/a	4.11 (2.01)	3.58 (1.91)	5.08 (1.47)	3.64 (2.27)	n/a	**0.010**	n/a	

Significant associations (*P* < 0.05) are bolded.

* Results of independent *t* test comparing pain-free group and chronic pain group combined (TMD + fibromyalgia + migraine).

† Results of ANOVA comparing the 3 chronic pain groups with each other.

‡ Results of ANCOVA comparing the 3 chronic pain groups with each other, controlling for pain intensity.

TMD, temporomandibular disorders.

Prescription medications were reported by 46.43% of the healthy control group, 80.77% of the TMD group, 100% of the fibromyalgia group, and 82% of the migraine group. The most common prescription medications across all groups included birth control medication, antihistamines, and antidepressants. Medication data by group are summarized in Table 3.

**Table 3:** Prescription medication use among young women with temporomandibular disorder, fibromyalgia, migraine, and pain-free controls.

	Pain-free group (N = 28)	TMD group (N = 26)	Fibromyalgia group (N = 25)	Migraine group (N = 25)
	n (%)	n (%)	n (%)	n (%)
No prescription medications	15 (53.57%)	5 (19.23%)	0 (0.00%)	3 (12.00%)
Prescription medication type				
Birth control/hormone medication	8 (28.57%)	7 (26.92%)	6 (24.00%)	7 (28.00%)
Allergy medication/antihistamines	3 (10.71%)	7 (26.92%)	10 (40.00%)	8 (32.00%)
Antidepressants	6 (21.43%)	16 (61.54%)	15 (60.00%)	14 (56.00%)
Stimulants	1 (3.57%)	4 (15.38%)	5 (20.0%)	3 (12.00%)
Muscle relaxants	0 (0.00%)	5 (19.23%)	1 (4.00%)	1 (4.00%)
Antiseizure/nerve medications	0 (0.00%)	3 (11.54%)	9 (36.00%)	2 (8.00%)
Cardiovascular medications, including alpha agonists and beta blockers	0 (0.00%)	2 (7.69%)	3 (12.00%)	2 (8.00%)
Migraine medications, including triptans, biologics, and injections	0 (0.00%)	5 (19.23%)	1 (4.00%)	9 (36.00%)
Diabetes medication	0 (0.00%)	2 (7.69%)	2 (8.00%)	0 (0.00%)
Immunosuppressants	0 (0.00%)	0 (0.00%)	2 (8.00%)	0 (0.00%)
Steroid medications	0 (0.00%)	0 (0.00%)	1 (4.00%)	0 (0.00%)
Thyroid medication	0 (0.00%)	5 (19.23%)	3 (12.00%)	2 (8.00%)
Prescription NSAIDS	0 (0.00%)	1 (3.85%)	5 (20.0%)	1 (4.00%)
Antacids/proton-pump inhibitors and other GI medications	0 (0.00%)	3 (11.54%)	6 (24.00%)	2 (8.00%)
Other prescription medication	0 (0.00%)	3 (11.54%)	4 (4.00%)	1 (4.00%)
Mean number of prescription medications per person	0.71 (SD = 0.85)	3.69 (SD = 3.90)	4.84 (SD = 3.79)	3.00 (SD = 2.38)

### 3.1. Comparison between the chronic pain group and pain-free controls

**Figure 1.**
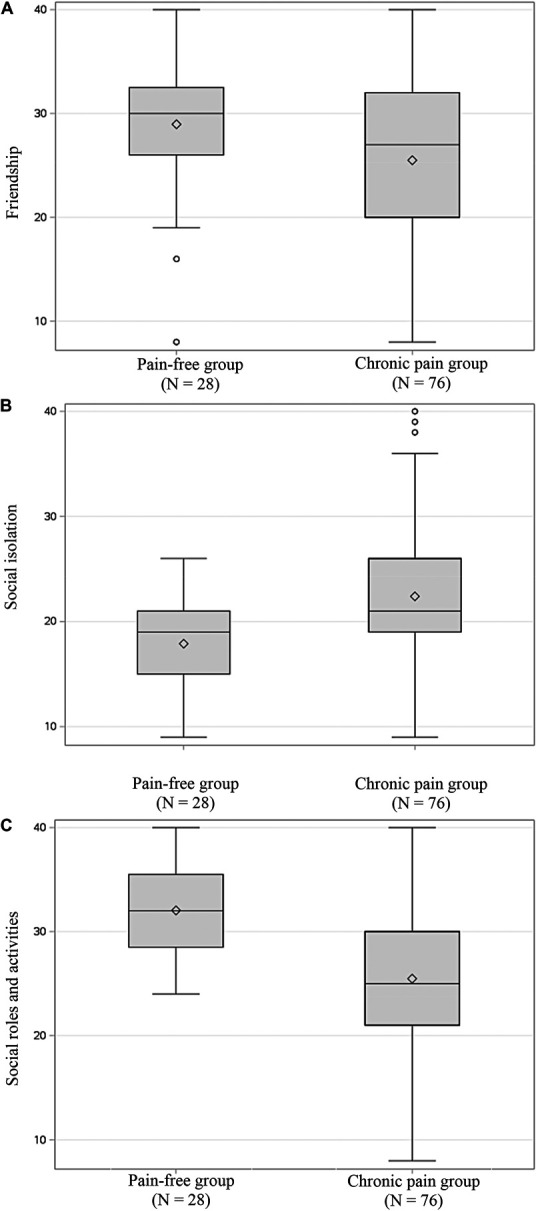
Raw scores for friendship (A), social isolation (B), and satisfaction with social roles and activities (C) between the pain-free group and the chronic pain group combined (TMD, fibromyalgia, and migraine). TMD, temporomandibular disorder.

All 3 chronic pain groups combined reported significantly worse functioning than pain-free controls on friendship (*P* = 0.038, Fig. 1A and Table 2), social isolation (*P* = 0.004, Fig. 1B and Table 2), and social roles (*P* < 0.001, Fig. 1C and Table 2), but did not differ on any other of the social constructs assessed.

### 3.2. Difference between the chronic pain conditions

Contrary to our hypothesis that young women with TMD would report worse social functioning than those with other chronic pain conditions, the 3 chronic pain conditions did not differ from one another on any social functioning variable (all *P*'s > 0.05) (Table 2). Although the average pain intensity significantly differed between the groups (*P* = 0.010), with participants experiencing fibromyalgia facing significantly worse pain compared with the TMD (*P* = 0.021, 95% CI −2.80, −0.19) and migraine groups (*P* = 0.026, 95% CI 0.15, 2.73), the 3 groups remained similar on social functioning even after controlling for pain intensity as a covariate (Table 2).

### 3.3. Chronic overlapping pain conditions

**Figure 2.**
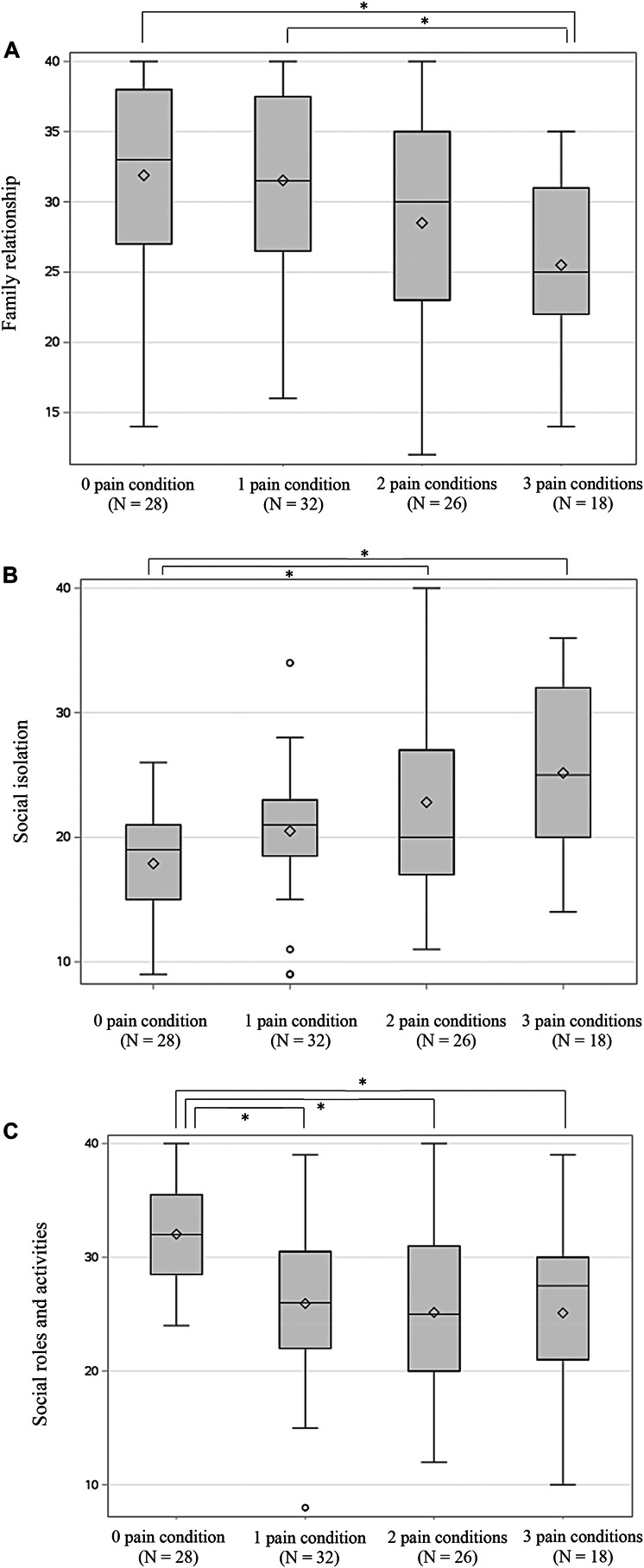
Raw scores for family relationship (A), social isolation (B), and satisfaction with social roles and activities (C) between the participants experiencing 0, 1, 2, or 3 chronic pain conditions (TMD, fibromyalgia, and migraine). TMD, temporomandibular disorder.

Of the 76 participants with chronic pain, 32 (42.1%) reported only 1 pain condition, 26 (34.2%) reported 2 conditions, and 18 (23.7%) reported 3 pain conditions. Pain-free controls reported better family relationships (*P* = 0.018, 95% CI 0.81, 11.98; see Table 4) than those with 3 chronic overlapping pain conditions. They also reported less social isolation than those with 2 (*P* = 0.020, 95% CI −9.27, −0.56) or 3 (*P* < 0.001, 95% CI −12.10, −2.45) chronic overlapping pain conditions and more satisfaction with their social roles and activities than patients with 1 (*P* = 0.002, 95% CI 1.76, 10.43), 2 (*P* = <0.001, 95% CI 2.32, 11.44), or 3 (*P* = 0.003, 95% CI 1.86, 11.98) chronic pain conditions. Those who reported 1 chronic pain condition exhibited significantly better family relationships than those with 3 chronic pain conditions (*P* = 0.024, 95% CI 0.59, 11.47; see Fig. 2). Age and income were similar between those with 0, 1, 2, or 3 pain conditions (*P* > 0.05). The remaining social domains did not differ as a function of the number of conditions (*P*'s > 0.05).

**Table 4:** Comparison of social functioning between young women with 0, 1, 2, or 3 chronic pain conditions.

	Healthy controls (ie, zero pain conditions) (N = 28)	1 pain condition (N = 32)	2 pain conditions (N = 26)	3 pain conditions (N = 18)	*P* comparing healthy controls with those with 1 pain condition	*P* comparing healthy controls with those with 2 pain conditions	*P* comparing healthy controls with those with 3 pain conditions	*P* comparing 1 pain condition with 2 pain conditions	*P* comparing 1 pain condition with 3 pain conditions	*P* comparing 2 pain conditions with 3 pain conditions
Social functioning										
Emotional support	34.64 (7.29)	34.56 (5.92)	33.08 (6.58)	34.39 (4.97)	0.99	0.80	0.99	0.81	0.99	0.91
Family relationships	31.89 (7.48)	31.53 (6.60)	28.50 (7.84)	25.50 (5.96)	0.99	0.30	**0**.**018**	0.37	**0.024**	0.51
Friendship	28.96 (7.29)	25.13 (6.30)	25.92 (7.04)	25.56 (10.21)	0.21	0.45	0.44	0.98	0.99	0.99
Informational support	33.82 (5.05)	33.47 (4.27)	31.77 (5.71)	31.33 (4.64)	0.99	0.43	0.35	0.56	0.46	0.99
Social isolation	17.89 (4.01)	20.50 (5.12)	22.81 (8.06)	25.17 (6.69)	0.36	**0.020**	**<0.001**	0.48	0.050	0.59
Social roles	32.04 (4.62)	25.93 (6.51)	25.15 (6.87)	25.11 (7.83)	**0.002**	**<0.001**	**0.003**	0.97	0.97	0.99
Hostility	14.04 (5.22)	15.03 (4.61)	16.58 (6.78)	16.06 (5.59)	0.90	0.35	0.63	0.72	0.92	0.99
Condition prevalence										
TMJD	NA	13 (40.6%)								
Fibromyalgia		16 (50.0%)								
Migraine		3 (9.4%)								
TMJD + fibromyalgia			10 (38.5%)							
TMJD + migraine			5 (19.2%)							
Fibromyalgia + migraine			11 (42.3%)							
TMD + fibromyalgia + migraine				18 (100%)						

Significant associations (*P* < 0.05) are bolded.

## 4. Discussion

Little is known about the associations between social parameters and pain in young adults. This is a critical gap in knowledge because young adulthood is characterized by important life transitions (eg, forming lifelong friendships, dating/getting married, starting a family, etc) that set the stage for functioning throughout the rest of adulthood. The goals of this study were (1) to compare which social parameters were different between young women with chronic pain and healthy controls; (2) test whether specific chronic pain conditions were differentially associated with social outcomes; and (3) test whether young women with multiple chronic pain conditions exhibited greater social burden than young women with only 1 chronic pain condition.

Results of our first aim suggest that not all aspects of social functioning are equally influenced by the experience of chronic pain. Parameters that differed between those with chronic pain and pain-free controls were friendships, social isolation, and satisfactions with one's social roles and activities. Social isolation has been previously documented in the adult chronic pain literature^3,26,37,38,64^ and may stem partly from the fact that people tend to report feeling stigmatized and lonely when they have “invisible” illnesses.^44^ The relationships between social isolation and chronic pain are likely bidirectional, where pain increases feelings of isolation and isolation exacerbates pain-related disability and intensity.^11,50^ That this difference between groups is already seen in a young adult sample is concerning and suggest that social isolation either happens early in the course of chronic pain or that it may be a risk factor of developing chronic pain in young adulthood. Our findings are consistent with existing research that also finds that chronic pain is associated with feelings of social isolation and disrupted social functioning in university students and adolescents with chronic pain^15,63^ and with qualitative research showing that young women with chronic pain report peer separation as a social impact of their pain.^74^ Future work should test these possibilities and their potential underlying mechanism.

Pain may affect satisfaction with social activities through several mechanisms. For example, chronic pain may exacerbate depression,^65^ negative affect,^22^ and fatigue^9,10,12^—each of which may limit one's willingness to participate in social leisure activities or alter perceptions of how enjoyable those activities are. If young women are avoiding participating in social activities because of their chronic pain or are dissatisfied with those activities, this may serve to perpetuate depression, anxiety, and loneliness that contribute to long-term pain disability in later adulthood.^26,33,37,46^ Thus, what may seem adaptive in the short-term (ie, avoiding social activities) may be maladaptive in the long-term, and interventions aiming to maintain social activities among young women experiencing chronic pain may be important. This is particularly important because evidence suggests social pain can also enhance physical pain.^23–25^

We did not find significant differences in social functioning between any of the 3 chronic pain conditions (TMD, fibromyalgia, and chronic migraine). This contrasted with our hypothesis that young women with TMD would report more social disruption than with other chronic pain conditions. We originally hypothesized this because TMD pain is often aggravated by innately social activities such as expressing emotions, talking, and eating (which is often done around other people). Yet, the social outcomes assessed in this study were not necessarily reliant on jaw activities. Thus, TMD may influence some domains of social functioning, such as communication and verbal expression, but not others, such as loneliness or family functioning. The specific social activities that may or may not be affected by TMD pain remain an important area for future research.

Even if there were no differences between the 3 chronic pain conditions examined, a growing body of literature is finding that the *number* of pain conditions one has is also an important predictor of functioning across a number of domains.^4,22,30,53,56,59,62^ To our knowledge, no one has previously examined whether social domains are also affected by chronic overlapping pain conditions among young women. We found that family relationships, social isolation, and satisfaction with social roles and activities were significantly better among those without chronic pain compared with those with 3 chronic pain conditions; those with 1 chronic pain condition also reported better family relationships than those with 3 chronic pain conditions. The reasons why chronic overlapping pain conditions resulted in worse outcomes on these specific domains remains unknown; we hypothesized similar relationships for all social domains. One possibility is that young women with chronic overlapping pain are spending more time isolating and as such they are more likely to be at home with their families as opposed to with friends. If this is the case, and if they are doing this when they are in the most pain, it is possible there is just more opportunity for familial life to be disrupted. However, more work is needed to formally test this question.

The study has significant limitations. Most critically, we used cross-sectional methodology, which is incapable of determining causal associations. Because we recruited using an online methodology (ie, ResearchMatch), selection bias may have affected our results. For example, it could be that participants with the worst social functioning would have been the ones most likely to participate in the research. All our data, including pain diagnosis data, were self-reported by participants. Moreover, because our primary aim was not to test differences in social functioning across multiple chronic overlapping pain conditions, we included only young women with chronic TMD, fibromyalgia, or migraines and thus had a limited range of all possible COPCs. Results may not generalize to young adults with chronic pain conditions other than the 3 included. Our sample sizes were small, and future work should replicate these findings in a larger sample of young women. Finally, our sample was almost entirely Caucasian. Thus, we were unable to detect any sex or race effects. Other research has identified the importance of both race and sex in various pain outcomes,^5,29,34^ and future work should replicate these findings in both sexes using a racially representative sample.

Despite these limitations, the study also has considerable strengths and clinical implications. To our knowledge, it is the only study to specifically focus on describing social outcomes among young women with chronic pain. Clinically, the findings highlight the importance of assessing social outcomes when working with this population and highlight the need for more clinical interventions aimed at reducing social isolation. Whereas studies have demonstrated favorable outcomes of chronic pain support groups and group-based cognitive therapies,^71^ few interventions, to our knowledge, are specifically designed to improve social function. Even if they do not specifically cover social topics, chronic pain support groups may exert some of their effects by promoting social bonding between members^7,41,42^ or promoting adaptive coping.^19^ Still, the effectiveness of such interventions may be even greater if they were designed to specifically address strategies for improving social isolation and satisfaction with social roles and activities. It is our hope that this study prompts future work uncovering how chronic pain affects social functioning among young women, so that better biopsychosocial interventions can be developed in the future.

## Disclosures

The authors have no conflict of interest to declare.
